# Estimates of number of children and adolescents without access to surgical care

**DOI:** 10.2471/BLT.18.216028

**Published:** 2019-01-28

**Authors:** Bhargava Mullapudi, David Grabski, Emmanuel Ameh, Doruk Ozgediz, Hariharan Thangarajah, Karen Kling, Blake Alkire, John G Meara, Stephen Bickler

**Affiliations:** a Division of Pediatric Surgery, Rady Children’s Hospital–University of California San Diego, 3030 Children's Way, San Diego, California, CA 92123, United States of America (USA).; b Department of Surgery, University of Virginia, Charlottesville, USA.; c Department of Surgery, National Hospital, Abuja, Nigeria.; d Division of Pediatric Surgery, Yale University, New Haven, USA.; e Program in Global Surgery and Social Change, Harvard Medical School, Boston, USA.

## Abstract

**Objective:**

To estimate how many children and adolescent worldwide do not have access to surgical care.

**Methods:**

We estimated the number of children and adolescents younger than 19 years worldwide without access to safe, affordable and timely surgical care, by using population data for 2017 from the United Nations and international data on surgical access in 2015. We categorized countries by World Bank country income group and obtained the proportion of the population with no access to surgical care from a study by the *Lancet* Commission on Global Surgery.

**Findings:**

An estimated 1.7 billion (95% credible interval: 1.6–1.8) children and adolescents worldwide did not have access to surgical care in 2017. Lack of access occurred overwhelmingly in low- and middle-income countries where children and adolescents make up a disproportionately large fraction of the population. Moreover, 453 million children younger than 5 years did not have access to basic life-saving surgical care. According to *Lancet* Commission on Global Surgery criteria, less than 3% of the paediatric population in low-income countries and less than 8% in lower-middle-income countries had access to surgical care.

**Conclusion:**

There were substantial gaps in the availability of surgical services for children worldwide, particularly in low- and middle-income countries. Future research should focus on developing specific measures for assessing paediatric surgical access, delivery and outcomes and on clarifying how limited surgical access in the poorest parts of the world affects child health, especially mortality in children younger than 5 years.

## Résumé

**Objectif:**

Estimer le nombre d'enfants et d'adolescents, à l'échelle mondiale, qui n'ont pas accès à des soins chirurgicaux.

**Méthodes:**

En utilisant les données démographiques de 2017 des Nations Unies et des données internationales de 2015 sur l'accès aux soins chirurgicaux, nous avons évalué le nombre d'enfants et d'adolescents de moins de 19 ans, à l'échelle mondiale, qui n'ont pas accès à des soins chirurgicaux sûrs, abordables et en temps opportun. Nous avons regroupé les pays en suivant la classification par niveau de revenu de la Banque mondiale, et nous avons calculé la proportion de la population n’ayant pas accès à des soins chirurgicaux à partir d'une étude réalisée par la Commission du *Lancet* sur la chirurgie dans le monde.

**Résultats:**

Environ 1,7 milliard (intervalle de crédibilité de 95%: 1,6–1,8) d'enfants et d’adolescents n'avaient pas accès à des soins chirurgicaux en 2017. Ce problème frappe de manière particulièrement écrasante les pays à revenu faible ou intermédiaire, où les enfants et adolescents constituent une part disproportionnellement importante de la population. Par ailleurs, 453 millions d'enfants de moins de 5 ans n'avaient pas accès à des interventions chirurgicales vitales de base. Selon les critères de la Commission du *Lancet* sur la chirurgie dans le monde, dans les pays à revenu faible et dans les pays à revenu intermédiaire, respectivement, moins de 3% et moins de 8% de la population pédiatrique avait accès à des soins chirurgicaux.

**Conclusion:**

Il existe des carences considérables en termes de disponibilité des soins chirurgicaux pour les enfants dans le monde, plus particulièrement dans les pays à revenu faible ou intermédiaire. De nouvelles recherches devraient être menées sur la conception de mesures spécifiques pour évaluer l'accès aux soins chirurgicaux pédiatriques, la prestation de ces soins et leurs résultats et sur la manière dont un accès limité aux soins chirurgicaux dans les régions les plus pauvres du monde affecte la santé des enfants, en particulier la mortalité des enfants de moins de 5 ans.

## Resumen

**Objetivo:**

Estimar cuántos niños y adolescentes en todo el mundo no tienen acceso a la atención quirúrgica.

**Métodos:**

Estimamos el número de niños y adolescentes menores de 19 años en todo el mundo sin acceso a una atención quirúrgica segura, asequible y oportuna, utilizando datos de población para 2017 de las Naciones Unidas y datos internacionales sobre el acceso quirúrgico en 2015. Clasificamos a los países por grupos de ingresos del Banco Mundial y obtuvimos la proporción de la población sin acceso a la atención quirúrgica de un estudio de la *Lancet* Commission on Global Surgery.

**Resultados:**

Se estima que 1.700 millones (intervalo creíble del 95 %: 1,6-1,8) de niños y adolescentes en todo el mundo no tenían acceso a la atención quirúrgica en 2017. La falta de acceso se produjo abrumadoramente en los países de ingresos bajos y medios, donde los niños, niñas y adolescentes constituyen una fracción desproporcionadamente grande de la población. Además, 453 millones de niños menores de 5 años no tienen acceso a la atención quirúrgica básica que salva vidas. Según los criterios de la *Lancet* Commission on Global Surgery, menos del 3 % de la población pediátrica de los países de bajos ingresos y menos del 8 % de los países de ingresos medios-bajos tenían acceso a la atención quirúrgica.

**Conclusión:**

En todo el mundo había considerables diferencias en la disponibilidad de servicios quirúrgicos para los niños, en particular en los países de ingresos bajos y medios. Las investigaciones futuras deben centrarse en el desarrollo de medidas específicas para evaluar el acceso quirúrgico pediátrico, la natalidad y los resultados, y en aclarar cómo el acceso quirúrgico limitado en las partes más pobres del mundo afecta la salud infantil, especialmente la mortalidad en niños menores de 5 años.

## ملخص

**الغرض:**

لتقدير عدد الأطفال والمراهقين في جميع أنحاء العالم والذين لا يحصلون على رعاية جراحية.

**الطريقة:**

لقد قمنا بتقدير عدد الأطفال والمراهقين الذين تقل أعمارهم عن 19 عاماً في جميع أنحاء العالم والذين لا يحصلون على رعاية جراحية آمنة وبأسعار معقولة وفي الوقت المناسب، وذلك باستخدام البيانات السكانية لعام 2017 من الأمم المتحدة والبيانات الدولية حول الحصول على الرعاية الجراحية عام 2015. قمنا بتصنيف البلدان حسب مجموعة دخل الدول من البنك الدولي وحصلنا على نسبة عدد السكان الذين لا يحصلون على الرعاية الجراحية من دراسة أجرتها لجنة لانسيت في الجراحة العالمية.

**النتائج:**

ما يقدر بحوالي 1.7 مليار طفل ومراهق (95% من الفترات ذات المصداقية: 1.6 - 1.8) في جميع أنحاء العالم لم يتمكنوا من الحصول على الرعاية الجراحية عام 2017. وقد حدث نقص كبير في الوصول إلى الخدمات في البلدان منخفضة ومتوسطة الدخل، حيث يشكل الأطفال والمراهقون جزءاً كبيراً غير متناسب من السكان. علاوة على ذلك، فإن 453 مليون طفل دون سن الخامسة لم يتمكنوا من الحصول على الرعاية الجراحية الأساسية المنقذة للحياة. ووفقاً لمعايير لجنة لانسيت العالمية للجراحة، فإن أقل من 3% من عدد الأطفال في البلدان منخفضة الدخل وأقل من 8% في البلدان ذات الدخل المتوسط الأدنى حصلوا على الرعاية الجراحية.

**الاستنتاج:**

كانت هناك فجوات كبيرة في توافر الخدمات الجراحية للأطفال في جميع أنحاء العالم، ولا سيما في البلدان منخفضة ومتوسطة الدخل. يجب أن يركز البحث المستقبلي على تطوير تدابير محددة لتقييم حصول الأطفال على الرعاية الجراحية وتقديم الخدمة والنتائج، وتوضيح مدى محدودية الحصول على الرعاية الجراحية في أفقر أجزاء العالم التي تؤثر على صحة الطفل، خاصة معدل وفيات الأطفال دون سن الخامسة.

## 摘要

**目的:**

旨在估计全球无法获得外科护理的儿童和青少年人数。

**方法:**

我们使用了 2017 年联合国人口统计数据和 2015 年全球获得外科护理人数的数据，估算出全球无法获得安全、经济实惠与及时外科护理的 19 岁以下儿童和青少年人数。我们根据世界银行国家收入分组对国家进行分类，并从《柳叶刀》全球外科委员会的一项研究中获取了无法获得外科护理的人口比例。

**结果:**

估计全球有 17 亿（95% 置信区间：1.6-1.8）儿童和青少年在 2017 年无法获得外科护理。在低收入和中等收入国家，无法获得外科护理服务的儿童和青少年占较大比例，形势较为严峻。此外，无法获得基本救生外科护理的 5 岁以下儿童人数为 4.53 亿。根据《柳叶刀》全球外科委员会的标准，在低收入国家，获得外科护理的儿童族群不足 3%，而在中低收入国家，获得外科护理的儿童族群不足 8%。

**结论:**

针对儿童的外科护理服务在世界范围内存在着巨大差异，尤其是在低收入和中等收入国家。今后的研究应侧重于制定评估获取儿科手术、分娩和并发症的具体措施，并阐明在世界上最贫穷的地区，获取外科护理受限对儿童健康，特别是对 5 岁以下儿童死亡率的影响。

## Резюме

**Цель:**

Оценка количества детей и подростков во всем мире, не имеющих доступа к хирургической помощи.

**Методы:**

Авторы оценили количество детей и подростков младше 19 лет во всем мире, не имеющих доступа к безопасной, доступной и своевременной хирургической помощи, опираясь на демографические данные ООН за 2017 год и международные данные о доступе к хирургической помощи за 2015 год. Страны были разделены по группам доходов согласно определению Всемирного банка, после чего авторы рассчитали долю населения, не имеющего доступа к хирургической помощи, на основании исследования, проведенного Комиссией по глобальной хирургии журнала *Lancet*.

**Результаты:**

Примерно 1,7 миллиарда детей и подростков (95%-й ДИ: 1,6–1,8) во всем мире не имели доступа к хирургической помощи в 2017 году. В абсолютном большинстве случаев такой недостаток доступа характерен для стран с низким и средним уровнем дохода, где дети и подростки составляют непропорционально большую долю населения. Кроме того, 453 миллиона детей младше 5 лет не имели доступа к базовой хирургической помощи, необходимой для спасения жизни. Согласно критериям Комиссии по глобальной хирургии журнала *Lancet*, менее 3% детского населения в странах с низким уровнем дохода и менее 8% в странах с уровнем дохода ниже среднего имеют доступ к хирургической помощи.

**Вывод:**

В мировом масштабе имеются значительные пробелы в доступности хирургической помощи для детей, в частности в странах с низким и средним уровнем дохода. В будущем необходимо сосредоточить усилия исследователей на разработке особых мер для оценки доступности детской хирургической помощи, практики оказания таких услуг и их исходов, а также на прояснении взаимосвязи между ограниченным доступом к хирургической помощи в беднейших странах мира и здоровьем детей, особенно как он влияет на смертность детей младше 5 лет.****

## Introduction

The Millennium Development Goal period (i.e. 2000 to 2015) was characterized by an unprecedented decrease in child mortality.^1^ Even in the poorest areas of the world, mortality in children younger than 5 years fell dramatically, which led to predictions that a grand global convergence in mortality in this age group would be possible by 2035.^2^ However, further progress will depend on continued improvements across the full spectrum of child health services.

Surgical care for children is one area of child health that is often overlooked, yet can play an important role in preventing death and disability.^3^ Surgery is vital for the repair of correctable congenital anomalies (e.g. congenital heart disease, cleft lip and palate and club foot), the treatment of life-threatening injuries and burns, and the diagnosis and treatment of childhood cancers. Surgery can minimize the acute and long-term suffering of children, protect families from substantial financial loss and increase economic productivity. In addition, surgical care can play a role in achieving health-related sustainable development goals and targets, in particular: (i) ending preventable deaths in newborn babies and children younger than 5 years; (ii) reducing death and disability due to road traffic injuries and noncommunicable diseases; (iii) ensuring universal health coverage; and (iv) increasing the health workforce.^4^

In 2015, the *Lancet* Commission on Global Surgery reported that at least 4.8 billion people worldwide lacked access to surgical care.^5^^,^^6^ The Commission assessed the availability of surgical services using a chance tree, probability model, in which access was evaluated over four dimensions: (i) timeliness, which was assessed from the proportion of people with serious injuries who were transported by ambulance; (ii) surgical capacity, which was defined as the proportion of surgical procedures needed to meet demand that were actually undertaken; (iii) safety, which was assessed from the proportion of operating theatres with pulse oximetry; and (iv) affordability, which was defined as the proportion of patients undergoing surgery who were protected from catastrophic expenditure due to out-of-pocket payments.^6^ Access to surgical care varied widely across geographical regions, with more than 95% of the population in South Asia and central, eastern and western sub-Saharan Africa having no access. It was not clear, however, how lack of access affects the paediatric population. Consequently, in our analysis, we sought to answer the specific question: “How many children and adolescents worldwide lack access to safe, affordable and timely surgical care?”

## Methods

To estimate the number of children and adolescents without access to surgical care we used population data for 2017 from the United Nations and previously reported data on access to surgery from the *Lancet* Commission on Global Surgery.^5^^–^^7^ The proportion of the population without access to surgical care in countries in different World Bank income groups and their associated 95% credible intervals (CrIs) were extracted from Table 1 of Alkire et al.’s 2015 paper.^6^ Our analysis did not attempt to estimate the number of children and adolescents who required surgical care; instead we estimated the number that did not have access to surgical services had they been needed.

**Table 1:** Estimated number of children and adolescents worldwide without access to surgical care, by age and country income group, 2017

World Bank country income classification	Total population, in millions^a^	No. of children and adolescents, in millions (% of total population)^a^					Proportion of total population with no access to surgical care(95% CrI)^b^	No. of children and adolescent with no access to surgical care, in millions (95% CrI)				
		0–4 years	5–9 years	10–14 years	15–19 years	< 19 years		0–4 years	5–9 years	10–14 years	15–19 years	< 19 years
High	1180.1	65.1 (5.5)	66.8 (5.7)	65.6 (5.7)	68.6 (5.8)	266.1 (22.6)	14.9 (12.2–17.5)	9.7 (7.9–11.4)	10.0 (8.1–11.7)	9.8 (7.9–11.5)	10.2 (8.3–12.0)	39.6 (32.2–46.6)
Upper-middle	2588.4	185.2 (7.2)	177.1 (6.8)	171.0 (6.8)	174.0 (6.7)	707.3 (27.3)	58.7 (49.1–66.5)	108.7 (90.9–123.1)	104.0 (87.0–117.8)	100.4 (83.9–113.7)	102.2 (85.5–115.7)	415.2 (347.3–470.3)
Lower-middle	2969.9	319.8 (10.8)	308.8 (10.4)	295.1 (9.9)	281.4 (9.5)	1205.0 (40.6)	92.3 (89.3–94.5)	295.1 (285.5–302.2)	285.0 (275.7–291.8)	272.4 (263.5–278.9)	259.7 (251.3–265.9)	1112.2 (1 076.1–1138.7)
Low	641.9	103.4 (16.1)	91.5 (14.3)	80.5 (12.5)	69.5 (10.8)	344.8 (53.7)	97.7 (95.6–99.5)	101.0 (98.8–102.9)	89.4 (87.5–91.0)	78.6 (76.9–80.1)	67.9 (66.4–69.1)	336.9 (329.6–343.1)
**Total**	**7380.2**	**673.4 (9.1)**	**644.2 (8.7)**	**612.2 (8.3)**	**593.4 (8.0)**	**2523.2 (34.2)**	**67.3 (64.1–70.4)**	**453.2 (431.7–474.1)**	**433.6 (413.0–453.5)**	**412.0 (392.4–431.0)**	**399.4 (380.4–417.8)**	**1698.1 (1 617.4–1776.3)**

CrI: credible interval.

^a^ Population figures for 2017 were obtained from the United Nations.^7^

^b^ The proportion of the population with no access to surgical care was obtained from a 2015 *Lancet* Commission on Global Surgery study.^6^

Assuming the proxy markers used to assess access to surgical care in the *Lancet* Commission on Global Surgery’s study apply equally to children and adults, then the number of children and adolescents in a specific age and World Bank income group can be given by:
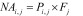
(1)where *NA_i,j_* is number of children and adolescents in age group *i* and World Bank income group *j* without access to surgical care, *P_i,j_* is the total population of children and adolescents in age group *i* and income group *j* and *F_j_* is the fraction of the total population in income group *j* without access to surgery.

The global population of children and adolescents without access to surgical care, *NA*, can then be determined by summing the estimated numbers in each age and income group:


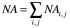
(2)

We estimated the number of children and adolescents without access to surgical care in the age groups 0 to 1, 1 to 4, 5 to 9, 10 to 14 and 15 to 19 years for each World Bank income category.

## Results

Table 1 shows: (i) the number of children and adolescents in different age groups worldwide in 2017, with countries grouped into the World Bank income categories of high, upper-middle, lower-middle and low income; (ii) surgical access rates reported in each income category by the *Lancet* Commission on Global Surgery study in 2015;^6^ and (iii) the estimated number of children and adolescents without access to surgery worldwide, categorized by age and country income group. We estimated that around 1.7 billion (95% CrI: 1.6–1.8) children and adolescents worldwide did not have access to surgical care in 2017. This figure corresponds to 67% (95% CrI: 64–70) of all children and adolescents worldwide. Lack of access occurred overwhelmingly in low- and middle-income countries, where 89.5% of individuals younger than 19 years live. Overall, 65% of the world’s children and adolescents without access to surgical care (i.e. 1.1 billion) live in lower-middle-income countries. In particular, 453 million children younger than 5 years did not have access to basic life-saving surgical care. When assessed using the *Lancet* Commission on Global Surgery’s criteria, less than 3% of the paediatric population living in low-income countries and less than 8% living in lower-middle-income countries had access to surgical care.

## Discussion

The World Health Organization has defined universal health coverage as the opportunity for any individual to have access to needed health services (including disease prevention, health promotion, treatment, rehabilitation and palliative care) of sufficient quality to be effective while also ensuring that use of these services does not result in financial hardship.^8^ Surgery and anaesthesia were officially recognized as indispensable components of universal health coverage in 2015 when the World Health Assembly adopted resolution WHA68.15.^9^ To facilitate implementation of the resolution, a growing number of countries are developing national surgery, obstetric and anaesthesia plans based on recommendations of the 3rd edition of the World Bank’s *Disease Control Priorities* and of the *Lancet* Commission on Global Surgery.^5^^,^^10^ These national plans involve an iterative process in which stakeholders use country-level data to develop contextually relevant and sustainable plans to ensure that surgical, obstetrical and anaesthetic services are available for an entire country or region.^11^ An appreciation of existing gaps in surgical services for children is important for ensuring that universal health coverage effectively encompasses the health-care needs of paediatric patients.

Although some might consider our estimate of the number of children and adolescents without access to surgical care to be too high, the number is consistent with our experience in many low-income countries, where surgical care for children has been a low priority and has often been excluded from child health programmes. Further, because children develop different surgical problems from adults and often require specialized care, the actual number without access could be even higher. The *Lancet* Commission used the availability of Caesarean section as a proxy marker for access. However, its availability does not necessarily ensure that the expertise, infrastructure and safety measures are in place to care for children with surgical problems. Indeed, a country could meet its targets for the number of surgical cases, health-care workforce and, even, financial protection without providing any surgical care for children. Specific metrics for paediatric surgical access are needed to help achieve equitable access to surgical and anaesthetic care for children.

It is important to emphasize that our study focused on access to surgical care and not the unmet need for care (i.e. when an individual has an untreated condition that would benefit from surgical care). Access refers simply to the availability of surgical care should it be needed. Unmet need would be a better metric because it could serve to quantify the impact of limited surgical care on child health. Nevertheless, it is difficult to see how the desired child health targets in low- and middle-income countries could be achieved when access to surgical care is so sparse. Surgical conditions are common in paediatric patients, with up to 85% of those younger than 15 years being affected.^12^ Consequently, poor access results in substantial morbidity and mortality.^13^ Moreover, the availability of surgical care is critical for infants as birth defects are now the fifth most common cause of death in children younger than 5 years.^14^ Without improvements in surgical and anaesthetic paediatric care, it will be impossible to achieve the second target of sustainable development goal 3: to end preventable deaths of newborns and children younger than 5 years by 2030.^15^

Finally, it is important to appreciate that the availability of surgical services does not necessarily ensure that a child or adolescent with a surgical condition will receive high-quality care. However, some elements of effectiveness were inherently considered in our analysis as the *Lancet* Commission on Global Surgery defined access as the availability of safe, affordable and timely surgical care. In future studies, other aspects of surgical delivery may need to be examined because the quality of care is also dependent upon the care structure (i.e. infrastructure, equipment and human resources for health), the process of care (i.e. the actual care delivered) and outcomes.^16^

In conclusion, we estimated that around 1.7 billion children and adolescents worldwide did not have access to surgical care in 2017. Most lived in low- and middle-income countries where children and adolescents make up a disproportionately large fraction of the population: less than 8% of the paediatric population in these countries has access to surgical care. Future priorities for research include developing specific metrics for assessing paediatric surgical access, delivery and outcomes, and clarifying how limited access to surgical care in the poorest regions of the world affects child health, especially mortality in children younger than 5 years. Efforts to scale up surgical care in low- and middle-income countries should consider the needs of children and adolescents.
